# Physical Disability and Psychedelic Therapies: An Agenda for Inclusive Research and Practice

**DOI:** 10.3389/fpsyt.2022.914458

**Published:** 2022-05-25

**Authors:** Kevin T. Mintz, Brinn Gammer, Amanda J. Khan, Gretchen Shaub, Steven Levine, Dominic Sisti

**Affiliations:** ^1^Stanford University, Center for Biomedical Ethics, Stanford, CA, United States; ^2^Department of Medical Ethics & Health Policy, University of Pennsylvania, Philadelphia, PA, United States; ^3^Department of Psychiatry, University of California, San Francisco, San Francisco, CA, United States; ^4^Sage Integrative Health, Berkeley, CA, United States; ^5^COMPASS Pathways, London, United Kingdom

**Keywords:** physical disability, sensory disability, psychedelic therapies, ethics, structural ableism in healthcare, justice, inclusion in clinical research

## Abstract

Over the past decade, there has been an increase in the number of clinical trials for psychedelic therapies as treatments for a wide range of psychiatric conditions. We are concerned that research organizations overseeing these trials have neglected the inclusion of individuals with physical and sensory disabilities. We suggest that psychedelic research organizations should prioritize and plan for the inclusion of individuals with physical and sensory disabilities to address the mental health burdens they confront. Not doing so risks reinforcing structural ableism in healthcare: the discriminatory manifestation of lowered expectations toward people with disabilities on the part of medical providers. Drawing on scholarship from disability studies and medical ethics, we offer four recommendations for disability inclusion in research. We recognize particular populations shoulder significant mental health burdens; these populations deserve priority and should be given a range of accommodations. We emphasize the need for extensive disability awareness training for those facilitating psychedelic therapies and encourage psychedelic researchers and therapists to exercise cultural humility toward individuals with physical and sensory disabilities. This article should be the impetus for further scholarship and debate about how psychedelic research and therapies can be made accessible to members of disability communities who might benefit.

## Introduction

In October 2021, the National Institutes on Drug Abuse announced that it would fund a clinical trial on the effects of psilocybin therapy and psychotherapy on smoking cessation (1). This announcement represents a milestone in the rebirth of research on psychedelics for the treatment of medical conditions, as it is the first federally funded study of its kind since the rescheduling and criminalization of psychedelics in the 1970s. Over the past 20 years, there has been a renewed recognition of the potential of classic psychedelics—including psilocybin, lysergic acid diethylamide (LSD), *N,N*-dimethyltryptamine (DMT), and mescaline—and empathogens like 3,4-Methylenedioxymethamphetamine (MDMA) to treat psychiatric and neurologic conditions. Significant amounts of private dollars have been invested in research programs aiming to bring several of these stereochemical isomers to market. Research on psychedelic medicines has thus expanded dramatically. In 2020, there were 17 clinical trials underway compared with zero only 10 years ago (2).

Psilocybin is currently under investigation for treating a range of conditions from treatment-resistant depression (3, 4) (TRD) to phantom limb pain (5). Phase 2 and 3 trials of MDMA-assisted therapy for posttraumatic stress disorder (PTSD) among civilians and veterans are underway (6–8). Mescaline, LSD, and DMT are being studied in the context of depression and anxiety (9). These studies build upon early signals which suggest psychedelic therapies may be legitimate alternatives to currently used—and marginally efficacious—treatments for these highly burdensome conditions. All this has fueled a new and rapidly expanding area of behavioral healthcare from which important and controversial ethical questions continue to emerge. These issues orbit the central topics of clinical and research ethics, including complexities related to informed consent, patient-clinician boundaries, and the identification and prevention of iatrogenic harms in the context of medical uncertainty. Recent cases of sexual boundary transgressions and abuse highlight the field-wide obligation to protect patients and carefully vet and hold accountable psychedelic researchers and therapists.

Research into psychedelic therapies also raises critical questions related to healthcare and social justice. These questions include determining who should be prioritized in receiving access to psychedelic research and therapies. Ethics and policy researchers have paid considerable attention to this question (10, 11). Answers to it ethically require recognizing both macroscopic inequities and the imperative of reciprocity to indigenous groups from whom researchers have gained early knowledge of these compounds. Ethical tensions continue to arise between ensuring equitable inclusion in psychedelic research and protecting community members from harm.

All of these challenges are relevant for the inclusion of a group that has largely been neglected in discussions of psychedelic therapies: people with disabilities. This group comprises individuals with a wide range of difficulties in various domains of functioning that ultimately affect one or more major life activities. Sixty-one million Americans and approximately one billion people worldwide live with some form of disability (12, 13).

The inclusion of people with disabilities in medical research is considered challenging for many reasons. A commonly cited concern is that many disabilities interfere with the ability to provide informed consent. Furthermore, many psychedelic studies exclude those with many underlying health conditions, making it less likely that people with disabilities will be able to participate. These barriers are not unique to the field of psychedelic research, but clinical research more generally (14–16).

In this paper, we argue that otherwise-qualified research participants with physical and sensory disabilities should not merely be considered eligible but, also, actively recruited into psychedelic studies as both an intrinsic matter of justice and to expand the evidence base for treatments that will soon be broadly available. Our argument is applicable to people whose conditions do not necessarily interfere with the capacity to provide informed consent but whose physical or sensory disabilities might pose substantial clinical or logistical obstacles during psychedelic therapies or for whom there may be additional considerations of potential adverse events. According to the American Community Survey for 2018, in the population of non-institutionalized adults aged 18 and over, an estimated 20,269,500 people have an ambulatory disability, 11,118,100 have a hearing disability, and 7,016,600 have a visual disability (17). The exclusions of these groups from research may be due to the reality that some disabling conditions are associated with comorbidities that disqualify an individual from research participation (3–9). For example, MDMA and psilocybin trials often exclude cardiac conditions or uncontrolled hypertension, because of the effect these psychedelics can have on resting heart rate and blood pressure (18–21). However, the logistical obstacles a physical or sensory disability might pose during a psychedelic therapy trial should not be a deterrent to conducting research with members of these communities, a sizeable percentage of whom confront significant mental health burdens.

## An Overlooked Population With Significant Mental Health Burdens

There have been a limited number of studies addressing the comorbidities of physical disability and mental health disorders such as major depressive disorder (MDD), anxiety disorders, and PTSD, among others. A limited amount of literature exists regarding these comorbidities; while some, such as Sareen et al. (22) consider correlation between PTSD and the development of short- and long-term disability, few examine the rates of mental disorders amongst disabled populations from the reversed perspective.

In assessing the need for advancements in mental health treatment for those with physical disabilities, we assembled evidence to ascertain in general terms psychiatric comorbidities with physical disability. Multiple sclerosis (MS), amyotrophic lateral sclerosis (ALS), cerebral palsy, muscular dystrophy, spinal cord injury, traumatic injury, deafness, blindness, and spinal muscular atrophy (SMA) were considered. Some searches yielded limited relevant results: there was only one study addressing comorbidities in the SMA population, for example (23). Data were only collected for children with separation anxiety disorder, phobia, ODD, PTSD, and MDD. Table 1 presents the community prevalence of several mental health disorders (24–32). Table 2 presents the prevalence of these disorders among individuals with particular physical disabilities (33–39).

**Table 1:** Community prevalence of mental disorders.

**Mental Illness**	**Adult population past year prevalence**
Major Depressive Disorder (40)	8.4%
Anxiety Disorders (24)	19.1%
Schizophrenia (25)	0.33%-0.75%.
Bipolar Disorder (26)	2.8%
Post-Traumatic Stress Disorder (27)	3.6%
Substance Use Disorders (31)	3.8%
Attention-Deficit/Hyperactivity Disorder (29)	4.4%
Bulimia Nervosa (30)	.3%
Personality Disorders (All) (32)	9.1%

**Table 2:** Disability community prevalence of mental disorders.

**Disabling condition**	**Prevalence within disability communities**	**Source**
MS	MDD - 46% Anxiety - 16.5% Bipolar - 2.4% Schizophrenia -.2%	Marrie RA, Horwitz R, Cutter G, Tyry T, Campagnolo D, Vollmer T, 2009 (33)
ALS	MDD - 17%	Zucchi E, Ticozzi N, Mandrioli J, 2019 (34)
Mild/Moderate Cerebral Palsy^a^	Anxiety Disorders - 19.5% Mood Disorders - 19.5% Schizophrenia - 2.8%	Whitney DG, Warschausky SA, Ng S, Hurvitz EA, Kamdar NS, Peterson MD, 2019 (35)^a^
Spinal cord injury	MDD - 20-30% Anxiety - 13.2–40% PTSD - 7.1–26.6%	Post M, van Leeuwen, C, 2012 (36)
Traumatic brain injury	MDD - 24.5% Anxiety - 24.5% Bipolar - ~1.6% Schizophrenia - 4.0% PTSD - 16.5% Substance Use Disorder - 11.5%	Rogers JM, Read CA, 2007 (37)
Deafness^b^	Anxiety Disorders - 18.7% Bipolar - 3.7% Impulse control disorders - 15.8% Substance Use Disorders 27.8% ADHD - 11.2%	Diaz DR, Landsberger SA, Povlinski J, Sheward J, Sculley C, 2013 (38)^b^
Blindness^c^	MDD - 18.2% Anxiety Disorders - 14.3% Schizophrenia - 1.4% Alcohol Misuse - 3.3% Psychoactive Substance Misuse - 12.9% Anorexia or Bulimia -.5%	Court H, McLean G, Guthrie B, Mercer SW, Smith DJ, 2014 (39)^c^

a Statistics in this table reflect the statistics for men with CP in this article.

b This article is focused on a population in outpatient psychiatric care.

c This article is focused on patients 65 and older in the UK.

The authors thank Jacob Kramer, an MSW student at Rhode Island College for helping bring these errors to our attention, particularly with regard to the D/deaf community.

There are considerable disparities between the prevalence of mental health disorders in physically healthy individuals and those with disabilities. Forty-seven percent of individuals with multiple sclerosis experienced MDD, in contrast to the general population prevalence of seven percent (33, 40). Similar differences exist across mental health disorders (for example, anxiety disorders, mood disorders, and schizophrenia were all reported more often in adults with cerebral palsy than in the general population) (35) as well as across physical disabilities (see Table 2). Considerations of equity and justice require that historically marginalized groups, including people with physical and/or sensory disabilities receive fair access to interventions that might benefit them. However, the mental health profession cannot fully begin to consider questions of broad and equitable access until research on psychedelic therapies sheds light on the effects of these treatments on those living with physical and/or sensory disabilities and whether they may benefit from them.

## How Neglecting Physical and Sensory Disabilities in Psychedelic Research Risks Reinforcing Structural Ableism in Healthcare

Disability communities have been historically discriminated against by medical professionals. For example, prior to the birth of the US disability rights movement in the 1970s, it was common for children and adults with disabilities to be institutionalized in state-run facilities with poor standards of health, safety, and quality of life (41). Even with the success of the disability rights and de-institutionalization movements, medical professionals express ignorance of disability rights legislation, such as the Americans with Disabilities Act (ADA), and discriminatory assumptions about the quality of life those with disabilities are capable of achieving. In a recent national survey of 714 physicians, Lisa Iezzoni and colleagues found that 82% of physicians believe that people with disabilities have a lower quality of life than those without disabilities (42). Furthermore, in a more recent paper, Iezzoni et al. report that 35.8% of the 714 physicians surveyed knew “little or nothing about their legal responsibilities under the ADA,” 71.2% further answered incorrectly about their responsibility to provide reasonable accommodations under the ADA, and 68.4% believed themselves to be at risk of an ADA lawsuit (43).

These data reflect broad misunderstandings about the concerns of disability communities among medical providers. Researchers in psychedelic therapies should be aware of this and other forms of structural ableism in healthcare, which is the discriminatory manifestation of lowered expectations toward people with disabilities on the part of medical providers. If people with physical and/or sensory disabilities are to be included in these trials, it may be helpful to note any history of medical discrimination as part of the intake before starting psychedelic interventions. Being aware of such discrimination can alert the therapist to features of the research setting or context that could be activating for a participant and use this as an opportunity to provide collaborative strengths-based corrective experiences.

Additionally, this research provides context for understanding what has been termed the disability paradox: the apparent disparity between how medical providers perceive the quality of life of patients with disabilities compared to how many disabled patients report having a similar or higher quality of life than non-disabled patients (44). This dichotomy in perspectives establishes the context for at least two different models of disability. The medical model posits that disability is a pathology worthy of treatment or cure. The social model of disability, by contrast, posits that disability is the result of an interaction between an impairment and inaccessible environments that limits opportunities for those living with their impairment (45).

In the context of psychedelic therapies, we propose that it is important to view disability from both perspectives. Those with physical and/or sensory disabilities may be seeking psychedelic therapy for the treatment of chronic pain or psychiatric comorbidities. At the same time, it would be discriminatory for the psychedelic research community to not take into account the social or environmental barriers that can make it harder for individuals with physical and/or sensory disabilities to participate in research. Neglecting these barriers would reinforce structural ableism in healthcare. To our knowledge, none of the existing studies of psychedelic therapies currently collect data on the coexistence of physical and/or sensory impairment with MDD, TRD, or PTSD. However, our concerns extend beyond issues of data collection.

In order to participate in trials for psychedelic therapies, people with physical and/or sensory disabilities will likely need a broad range of accommodations for various components of the therapeutic process. We use accommodations to describe a broad range of individualized adjustments necessary to provide disability access; this is broader than the legal definition of reasonable accommodations under the ADA, which only applies to adjustments for employees with qualifying health conditions (46). Wheelchair users will need to undergo therapy in spaces where furniture is arranged in such a way as to allow freedom of movement. Consent processes will need to be made accessible to those who require sign language interpreters or have communication difficulties. These accommodations are two straightforward examples. Depending on the complexity of a participant's physical and/or sensory impairments, any number of arrangements might need to be considered (e.g., prep session practice removing prostheses for those with amputations). People living with different disability diagnoses, or people with the same diagnosis but different levels of symptom severity, will most certainly require different kinds of accommodations. It is difficult to anticipate the entire range of these accommodations in the abstract. This reality underscores the need for psychedelic research organizations to engage with members of various disability communities so that researchers can proactively anticipate different needs among different populations.

It will be complicated to implement accommodations in psychedelic therapies due to the fact that the study design may have to be modified to integrate accommodations in a way that does not violate the scientific validity of the data being collected (e.g., considerations of music for participants who are deaf or hard-of-hearing). Moreover, disability accommodations often require additional financial, human, or other resources to implement. It is therefore important to engage in thoughtful dialogues, as well as organizational and financial planning about how to meet the ethical obligation to accommodate participants' disabilities. In what follows, we pose recommendations for how to begin these processes.

## Discussion

The gradual inclusion of people with physical and/or sensory disabilities in psychedelic research and clinical practice will require engagement across disability communities and the healthcare community. Our purpose has been to highlight the need for such engagement, but this article should be seen as only the beginning of a larger discussion. Here we present several modest proposals for how the discussion should move forward.

Recommendation #1: Psychedelic research groups should strive to meet and exceed the basic requirements of the Americans with Disabilities Act Amendments of 2008, Section 504 of the Rehabilitation Act of 1973, and other relevant disability rights legislation (46, 47). These laws have different standards and requirements for different entities, and all stakeholders in psychedelic research must understand what is legally required. Therefore, psychedelic research organizations should enlist individuals with expertise in disability law as team members, helping to oversee current and future trials for psychedelic therapies. It is important that the psychedelic research community consider these laws as providing minimum legal requirements. However, best practice for disability inclusion in psychedelic therapies will likely mean implementing measures beyond what the law requires, especially since it is a rapidly evolving field.

Recommendation #2: Psychedelic research groups should engage in direct dialogue with individuals within specific disability communities about their perceptions, concerns, and access barriers related to psychedelic therapies. A recent psychedelic trial provides initial guidance on how such engagement should take place. In a study of psilocybin safety and efficacy for depression, anxiety, and demoralization among older long-term AIDS survivor men, investigators solicited the input of expert community consultants, which benefited the “study design (intervention characteristics and outcome measures), execution (clinician training and participant recruitment), analysis, and dissemination” (48). Such expertise should be sought from members of disability communities. Our analysis in this article is grounded in the belief that people with disabilities should be included in all aspects of public life. While we think that psychedelic therapies may benefit individuals with disabilities, we do not pretend to know what the specific concerns of different disability communities would be. Soliciting input from members of disability communities can help ensure that concerns and access barriers are accounted for at each stage of the research process, making future psychedelic trials as accessible to those with disabilities as possible.

Based on the data presented above regarding mental health burdens for people with physical and/or sensory disabilities, we suggest the psychedelic research community focus on outreach to advocacy organizations for MS, ALS, and deaf or hard-of-hearing communities. Besides their considerable mental health burdens, engagement with these groups is important because their accommodation needs are likely to be substantial. Accommodations should be considered across all phases of research from design, recruitment, intake, through to the final follow-up assessment. If the psychedelic research community develops best practices for accommodations with these groups, it should be considerably easier to work with other groups that require accommodations in the future.

Recommendation #3: Psychedelic research organizations should begin to incorporate disability awareness into training programs for guides who facilitate psychedelic therapies. Disability inclusion requires ongoing work. Even while the psychedelic research community takes steps to understand the specific concerns of different disability communities, there are certain topics relevant across groups that should be incorporated into all training programs.

These training programs should include discussion of disability rights history in the United States and internationally, the medical and social models of disability, the disability paradox, and the ethical issues arising from disability inclusion in psychedelic therapies. Some of these issues include how to negotiate and obtain informed consent, how to work with the caregivers of participants with disabilities in psychedelic therapies, and protecting those with disabilities from harm and exploitation at the hands of psychedelic guides.

The importance of this latter topic is paramount. While anyone can become more vulnerable to harm and exploitation while under the influence of mind-altering substances, people with physical and/or sensory disabilities are particularly vulnerable because of their additional limitations. We raise this concern out of recognition that women with disabilities are at a much higher risk for sexual assault than women without disabilities (49, 50). Psychedelic research organizations, as well as independent practitioners, have received allegations of sexual misconduct against non-disabled participants in psychedelic trials on the part of guides (51). While it would be paternalistic and discriminatory to exclude people with physical and/or sensory disabilities from these trials due to fear of sexual abuse, the psychedelic community must take proactive steps to ensure that participants with disabilities will not be exploited.

Recommendation #4: The psychedelic research community should demonstrate cultural humility toward people with disabilities. Disability communities, along with communities of color and indigenous populations, have endured considerable injustices on the part of healthcare providers and medical researchers. Although we share the psychedelic research community's enthusiasm for how psychedelic therapies might transform mental health treatment, it is important that disability communities be approached with respect and dignity, and not be seen as “guinea pigs” for psychedelic researchers to experiment on based on the ableist assumption that individuals in these communities need to be “cured” or “fixed”. Being conscientious and transparent about intentions and goals is important for cultivating trust with disability communities. By working in partnership, the psychedelic research community can establish best practices that will promote access to psychedelic therapies for disability communities in a way that respects their autonomy and enables them to exercise greater control over their own mental health.

## Data Availability Statement

The original contributions presented in the study are included in the article/supplementary material, further inquiries can be directed to the corresponding author/s.

## Author Contributions

KM, BG, and DS co-wrote the first draft of this manuscript. AK, SL, and GS provided extensive editorial comments and suggestions. BG compiled the studies discussed on mental health comorbidities amongst individuals with physical and sensory disabilities, and constructed Tables 1, 2. All authors worked collaboratively to craft the recommendations presented in the discussion section of this article.

## Funding

KM is funded by a T32 grant (SPO #120347) in the Ethical, Legal, and Social Implications of Genetics and Genomics through the National Human Genome Research Institute of the National Institutes of Health. The ideas presented in this article are those of the authors and do not necessarily reflect the views of the National Institutes of Health, the Department of Health and Human Services, the US Public Health Service, or any other federal agency.

## Conflict of Interest

DS reports receiving consultation fees from COMPASS Pathways. SL and GS are employees of COMPASS Pathways. The remaining authors declare that the research was conducted in the absence of any commercial or financial relationships that could be construed as a potential conflict of interest.

## Publisher's Note

All claims expressed in this article are solely those of the authors and do not necessarily represent those of their affiliated organizations, or those of the publisher, the editors and the reviewers. Any product that may be evaluated in this article, or claim that may be made by its manufacturer, is not guaranteed or endorsed by the publisher.
