# Corrigendum: Physical disability and psychedelic therapies: an agenda for inclusive research and practice

**DOI:** 10.3389/fpsyt.2024.1523364

**Published:** 2024-12-23

**Authors:** Kevin T. Mintz, Brinn Gammer, Amanda J. Khan, Gretchen Shaub, Steven Levine, Dominic Sisti

**Affiliations:** ^1^ Stanford University, Center for Biomedical Ethics, Stanford, CA, United States; ^2^ Department of Medical Ethics & Health Policy, University of Pennsylvania, Philadelphia, PA, United States; ^3^ Department of Psychiatry, University of California, San Francisco, San Francisco, CA, United States; ^4^ Sage Integrative Health, Berkeley, CA, United States; ^5^ COMPASS Pathways, London, United Kingdom

**Keywords:** physical disability, sensory disability, psychedelic therapies, ethics, structural ableism in healthcare, justice, inclusion in clinical research

In the published article, there were errors in **Table 2**. The authors wish to correct the errors and clarify data presented in **Table 2**, pertaining to the prevalence of mental illness in several populations.

**Table 2:** Disability community prevalence of mental disorders.

Disabling condition	Prevalence within disability communities	Source
MS ALS Mild/Moderate Cerebral Palsy^a^ Spinal cord injury Traumatic brain injury Deafness^b^ Blindness^c^	MDD - 46% Anxiety - 16.5% Bipolar - 2.4% Schizophrenia -.2% MDD - 17% Anxiety Disorders - 19.5% Mood Disorders - 19.5% Schizophrenia - 2.8% MDD - 20-30% Anxiety - 13.2-40% PTSD - 7.1–26.6% MDD - 24.5% Anxiety - 24.5% Bipolar - ~1.6% Schizophrenia - 4.0% PTSD - 16.5% Substance Use Disorder - 11.5% Anxiety Disorders – 18.7% Bipolar – 3.7% Impulse control disorders – 15.8% Substance Use Disorders – 27.8% ADHD – 11.2% MDD - 18.2% Anxiety Disorders - 14.3% Schizophrenia - 1.4% Alcohol Misuse - 3.3% Psychoactive Substance Misuse - 12.9% Anorexia or Bulimia -.5%	Marrie RA, Horwitz R, Cutter G, Tyry T, Campagnolo D, Vollmer T, 2009 (33) Zucchi E, Ticozzi N, Mandrioli J, 2019 (34) Whitney DG, Warschausky SA, Ng S, Hurvitz EA, Kamdar NS, Peterson MD, 2019 (35)^a^ Post M, van Leeuwen, C, 2012 (36) Rogers JM, Read CA, 2007 (37) Diaz DR, Landsberger SA, Povlinski J, Sheward J, Sculley C, 2013 (38)^b^ Court H, McLean G, Guthrie B, Mercer SW, Smith DJ, 2014 (39)^c^

a Statistics in this table reflect the statistics for men with CP in this article.

b This article is focused on a population in outpatient psychiatric care.

c This article is focused on patients 65 and older in the UK.

The authors thank Jacob Kramer, an MSW student at Rhode Island College for helping bring these errors to our attention, particularly with regard to the D/deaf community.

Under “Mild/Moderate Cerebral Palsy”, the data in column 2 refer to men only. A footnote has been added to clarify this.

Under “Spinal cord injury”, the data in column 2 were reported as 7.1–26.6% and 13.2-40% for Anxiety and PTSD, respectively. The correct values are 13.2–40% and 7.1–26.6%. The table has been updated to reflect this.

Under “Deafness”, the data for Substance Use Disorder in column 2 reflect a population in outpatient psychiatric care. A footnote has been added to clarify this.

Also under “Deafness”, the data in column 2 were reported as 30.1%, 14.2%, 5.2%, 48.4% and 4.9% for Anxiety, Bipolar, Impulse control disorders, Substance Use Disorders and ADHD, respectively. The correct values are 18.7%, 3.7%, 15.8%, 27.8% and 11.2%. The table has been updated to reflect this.

Under “Blindness”, the data in column 2 refer to patients older than 65. A footnote has been added to clarify this.

The corrected **Table 2** and its caption appear below.

The authors apologize for these errors and state that this does not change the scientific conclusions of the article in any way. The original article has been updated.

